# Perceived Stress Profiles Among Italian University Students: A Multivariate Approach

**DOI:** 10.3390/healthcare13222830

**Published:** 2025-11-07

**Authors:** Valentina Micheluzzi, Elena Sandri, Anna Marchetti, Anna De Benedictis, Giorgia Petrucci, Rosaria Alvaro, Maria Grazia De Marinis, Michela Piredda

**Affiliations:** 1 Department of Biomedicine and Prevention, University of Rome Tor Vergata, Via Montpellier, 1, 00133 Rome, Italy; valentina.micheluzzi@gmail.com (V.M.);; 2 Clinical and Interventional Cardiology, University Hospital of Sassari, 07100 Sassari, Italy; 3 Faculty of Medicine and Health Sciences, Catholic University of Valencia San Vicente Mártir, c/Quevedo, 2, 46001 Valencia, Spain; elena.sandri@ucv.es; 4 Research Unit Nursing Science, Department of Medicine and Surgery, University Campus Bio-Medico di Roma, Via Alvaro del Portillo, 21, 00128 Rome, Italym.piredda@unicampus.it (M.P.); 5 Research Unit of Nursing Palliative Care, Fondazione Policlinico Universitario Campus Bio-Medico, Via Alvaro del Portillo, 200, 00128 Rome, Italy; 6 Research Unit of Orthopaedic and Trauma Surgery, Fondazione Policlinico Universitario Campus Bio-Medico, Via Alvaro del Portillo, 200, 00128 Rome, Italy

**Keywords:** academic stress, mental health, stress, university students, Italy

## Abstract

**Highlights:**

**What are the main findings?**

- Italian university students show moderate–high perceived stress; academic demands are the predominant stressors.
- Higher stress is observed in women, younger and first-year students, and those in Health Sciences/STEM, whereas lower stress/better coping is observed in older and postgraduate students and in Southern/Islands; private universities show higher academic pressure.

**What are the implication of the main findings?**

- Use early screening with validated instruments such as the IPSS-R to identify at-risk groups and provide targeted interventions.
- Implement institutional measures (workload/assessment review, counseling, peer support), prioritizing first-year and high-pressure programs.

**Abstract:**

**Background**: Perceived stress occurs when environmental demands are appraised as exceeding an individual’s coping resources, triggering emotional dysregulation and physiological hyperactivation with adverse mental and physical outcomes. University students are particularly vulnerable to psychological distress due to academic pressure, social transitions, and lifestyle changes. Despite increasing attention to mental health in higher education, data on perceived stress among Italian university students remain limited. This study aimed to assess stress profiles using the Italian Perceived Stress Scale—Revised (IPSS-R) and to explore associations with sociodemographic and academic variables. **Methods**: A multicenter cross-sectional study was conducted among 2.103 undergraduate and master’s students enrolled in Italian universities. Participants completed the 15-item IPSS-R, which measures three dimensions: general stress, coping, and academic stress. Sociodemographic and academic data were collected via a structured questionnaire. Non-parametric tests and Principal Component Analysis were employed to identify group differences and multivariate patterns. **Results**: Two principal components were retained through the principal component analysis, overall perceived stress (40.9% of the variance) and coping-related responses (13.7% of the variance). The mean total IPSS-R score was 30.6 (SD = 7.08, *p* < 0.001), reflecting moderate-to-high levels of perceived stress. Academic demands emerged as the predominant stressors. Higher stress levels were reported by female students, younger individuals, first-year undergraduates, and those enrolled in health sciences and STEM programs. Conversely, older students, postgraduate students, and those studying in Southern Italy demonstrated stronger coping abilities and lower academic stress. Students attending private universities reported elevated academic pressure, potentially due to heightened family and financial expectations. **Conclusions**: Italian university students experience substantial perceived stress, primarily driven by academic workload, performance expectations, and institutional pressure. Early identification using instruments such as the IPSS-R may enable targeted interventions to promote mental health and academic achievement in student support services, during triage, in wellness checks, and in psychoeducation.

## 1. Introduction

Perceived stress is a multifaceted psychological construct that reflects the subjective appraisal of life circumstances as unpredictable, uncontrollable, and overwhelming—rather than a direct reflection of objective stressors. This concept is rooted in Lazarus and Folkman’s (1984) [1] transactional model of stress and coping, which posits that stress arises when individuals perceive environmental demands as exceeding their adaptive resources. Such perceptions elicit specific cognitive, emotional, behavioral, and physiological responses [1]. According to this framework, perceived stress is shaped not solely by exposure to stressful events, but by the individual’s cognitive appraisal of those events. Stress arises when a situation is perceived as threatening and the individual believes they lack adequate resources to cope with it [2]. When demands surpass perceived coping capacity, this appraisal triggers emotional dysregulation—manifested as heightened anxiety, negative affect, and reduced emotional control—and activates neurophysiological stress pathways. Specifically, the stress response involves hyperactivation of the hypothalamic–pituitary–adrenal (HPA) axis and the sympathetic–adrenal–medullary system, resulting in increased secretion of cortisol and catecholamines [3,4]. While these responses are adaptive in acute situations, chronic or repeated activation becomes maladaptive, contributing to structural and functional brain changes such as hippocampal atrophy and prefrontal cortex dysregulation. These alterations are associated with long-term mental health consequences, including anxiety disorders and depression [5,6]. Furthermore, sustained neuroendocrine activation disrupts cardiovascular and immune homeostasis by promoting endothelial dysfunction, increasing pro-inflammatory cytokines, and impairing immune cell activity. These effects increase susceptibility to cardiovascular diseases, malignancies, and infections. These physiological responses contribute to emotional and cognitive symptoms observed in stressed students [7,8].

A critical developmental period for stress vulnerability is the transition to university, characterized by separation from established support networks [9,10], adaptation to demanding academic environments, and financial or occupational challenges [11]. These factors can exacerbate perceived stress [1]. Research indicates that diminished social support—whether familial, peer-based, or institutional—is associated with elevated stress levels and poorer mental health outcomes [12]. Financial and occupational stressors, including tuition fees, housing expenses, and part-time employment, further compound this vulnerability by limiting coping resources and exacerbating the subjective experience of stress [13,14]. First-year university students are particularly susceptible to perceived stress due to the abrupt shift from secondary school to university life [15]. This transition entails significant adjustments to academic expectations, social contexts, and often a weakening of familiar support networks. These challenges have been consistently linked to heightened anxiety, depressive symptoms, and burnout, all of which adversely affect mental health and academic performance [16]. Additionally, high academic workloads, perfectionistic tendencies, and performance-related pressures further exacerbate stress, increasing the risk of persistent psychological distress [17,18]. Academic stress has also been shown to impair sleep quality, emotional regulation, and cognitive functioning, thereby compromising students’ well-being and academic success [19].

The cultural and educational characteristics of Italian universities may also influence students’ experiences of stress. Competitive entry processes, concentrated exam sessions with potential delays in academic progression, administrative deadlines, high housing costs, limited infrastructure, long-distance relocation—particularly from Southern regions—and mandatory internships often involving indirect costs may all heighten students’ stress [20,21].

Although research on perceived stress among university students in Italy is growing, many studies have relied on small or localized samples, underscoring the importance of recent multicenter efforts [20].

Furthermore, few investigations have employed systematic multivariate analyses to explore the interplay between sociodemographic and academic variables.

The study aimed to (1) investigate perceived stress profiles among Italian university students; (2) examine associations between sociodemographic characteristics, general stress, academic stress-related experiences, and coping capacity; (3) explore the underlying structure of stress-related variables and their potential links to sociodemographic factors; and (4) assess how stress-related experiences vary across different sociodemographic profiles.

## 2. Materials and Methods

### 2.1. Study Design

This research employed a multicenter, cross-sectional observational design. This design enabled us to capture regional and institutional variability, providing a broader and more representative assessment of stress profiles among Italian students.

### 2.2. Instrument

Data were collected using the Italian Perceived Stress Scale—Revised (IPSS-R), an adaptation of the widely used Perceived Stress Scale (PSS) [6,22] recently validated for Italian university students [23]. The IPSS-R includes 15 items rated on a 5-point Likert scale (0–4), grouped into three subscales: Perceived Stress, Coping, and Academic Stress (see Supplementary File, Table S1). Of the 15 items, 11 are negatively worded, while the four Coping items are positively phrased and reverse-coded so that higher scores across all subscales indicate greater perceived stress and difficulty coping. The omega values for Stress, Coping, and Academic Stress were 0.85, 0.78, and 0.79, respectively. The global reliability index and the Omega H for the entire scale were 0.74 and 0.76, respectively.

The survey also collected sociodemographic and academic data, including sex, age, geographic area, academic discipline, level of study, university type (public or private), offsite study status, religious affiliation, and practice, among others. Age was categorized into three groups: Young (18–24 years), Young adults (25–39 years), and Middle Age (40–62 years).

### 2.3. Sample and Data Collection

Convenience sampling was conducted via institutional mailing lists from universities across northern, central, and southern Italy, including the major islands, as well as through personal contacts of the research team. Eligible participants were undergraduate and master’s students aged 18 years or older enrolled in Italian universities. Data collection occurred online between January 2024 and March 2025 following a multicenter dissemination strategy. The study adhered to the STROBE guidelines for observational research [24].

### 2.4. Ethical Considerations

The study was conducted in accordance with the ethical principles of the Declaration of Helsinki [25]. The relevant Ethics Committees approved the study (Protocol 75.23 CET2-CBM, 26 October 2023). Participant confidentiality and data protection were ensured in compliance with current regulations. Informed consent was provided online prior to survey completion. This study was not preregistered.

### 2.5. Statistical Analyses

Initial data pre-processing involved removing invalid, inconsistent, or extreme entries. A total of 127 cases were excluded due to incomplete or inconsistent responses, leaving 2103 valid cases for analysis. Data cleaning procedures included detecting contradictory answers (e.g., simultaneously reporting very high stress and very high coping), missing key sociodemographic information, and implausible response patterns. Extreme values were inspected using z-scores (>±3 SD) and boxplots. Only clearly erroneous or inconsistent entries were removed to preserve data variability and maintain a representative sample.

The Shapiro–Wilk test revealed non-normal distributions for all variables, confirmed visually via Q–Q plots generated using DATAtab’s Online Statistics Calculator. Given the non-normality, non-parametric tests were applied where appropriate [26]: Chi-square test for categorical variables, and Mann–Whitney U or Kruskal–Wallis tests for group comparisons. These tests were used to statistically confirm patterns initially identified through visual inspection of principal component analysis (PCA) plots.

PCA was conducted to explore the underlying structure of stress-related variables and their potential associations with sociodemographic characteristics. As an unsupervised multivariate technique, PCA reduces dimensionality by transforming correlated variables into principal components that retain most of the variance. PCA is a linear transformation based on the variance–covariance (or correlation) structure of the dataset and can be appropriately applied to non-normally distributed data, particularly when the objective is data reduction or exploration of underlying patterns rather than to make inferential statements [27,28]. Variable biplots illustrated component loadings, while group plots visualized participant distribution across PCA space. In these plots, each point represents an individual participant. Points are color-coded by sociodemographic groups (e.g., sex, age categories) to visually identify clustering or separate patterns. All analyses were performed using Jamovi software (Version 2.6.26), with additional visualizations and figure refinements created in Microsoft Excel.

### 2.6. Transparency and Openness

The data, materials, and analysis code used in this study are not publicly available due to the sensitive nature of the data. However, they may be made available upon reasonable request to qualified researchers by contacting the corresponding author.

## 3. Results

### 3.1. Sample Description

Table 1 summarizes the sociodemographic profile of the 2103 university students. Most participants were female (76.2%) and enrolled in health sciences programs (70.2%), with the majority aged 18–24 years. Students were represented across all academic years and Italian regions, predominantly from Central Italy. Most attended public universities (81.6%), with 35.8% classified as offsite students and 19.4% receiving scholarships or places in Colleges of Merit. Regarding religion, 52.4% identified as Catholic, 27.2% as atheist or agnostic, and only 28.9% reported active religious practice.

### 3.2. Stress and Coping Profiles

**Figure A1:**
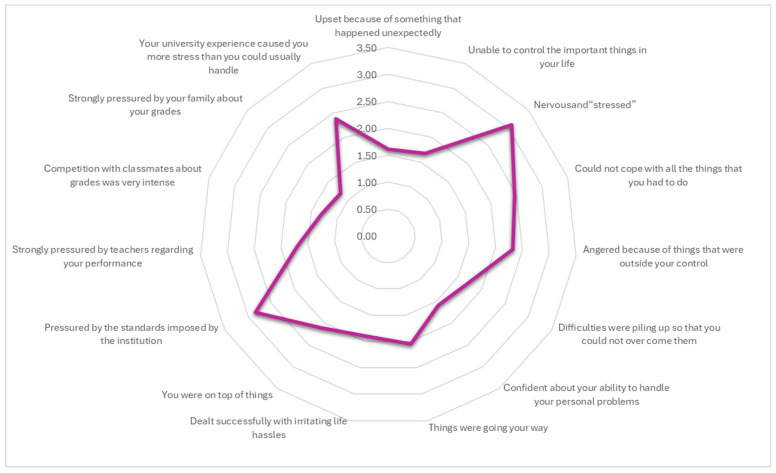
Graphical representation of IPSS-R item scores (N = 2103).

Table 2 and Figure A1 in Appendix A summarize the stress-related experiences reported by university students. The table shows mean scores and standard deviation for each item, as well as for the three IPSS-R dimensions and the overall scale. Figure A1 in Appendix A provides a graphical representation of these findings.

Among individual items, “Nervous and stressed” yielded the highest mean score (M = 3.08, SD = 0.91), indicating that emotional strain is a common experience among students. Academic stressors were also prominent, particularly the “Pressured by the standards imposed by the institution” (M = 2.84, SD = 1.11) and “Could not cope with all the things that you had to do” (M = 2.47, SD = 1.05). In contrast, interpersonal stressors such as “Strongly pressured by your family about your grades” (M = 1.18, SD = 1.31) and “Competition with classmates” (M = 1.30, SD = 1.32) scored notably lower, suggesting these are less commonly perceived as major stress factors. Items related to control and coping, including “Things were going your way” (M = 2.05, SD = 0.90) and “Dealt successfully with irritating life hassles” (M = 1.90, SD = 0.89), were rated moderately, indicating a partial capacity among students to manage stress effectively.

Across the three IPSS-R dimensions, the stress component had the highest mean score (M = 2.15, SD = 0.81), followed by difficulty in coping (M = 1.91, SD = 0.73) and academic stress (M = 1.88, SD = 0.89). The overall IPSS-R score averaged 29.9 (SD = 9.94), reflecting a substantial level of perceived distress within the sample. These results highlight the prevalence of general emotional stress, with academic pressures serving as an additional source of strain. While students demonstrate some coping ability, the data suggest a need for targeted interventions focused on emotional regulation, workload management, and resilience-building strategies.

**Figure 1:**
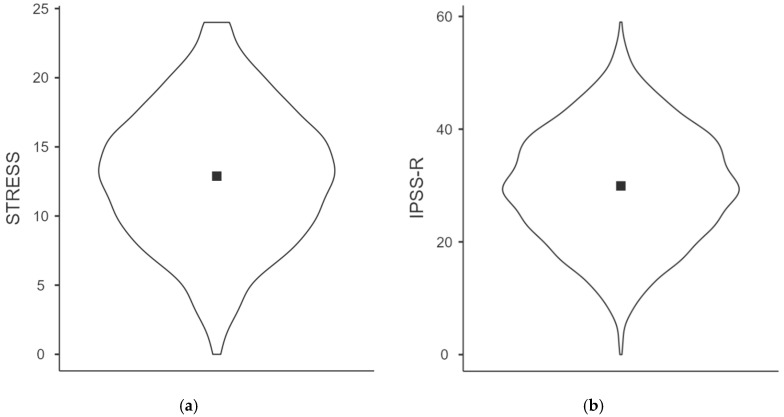

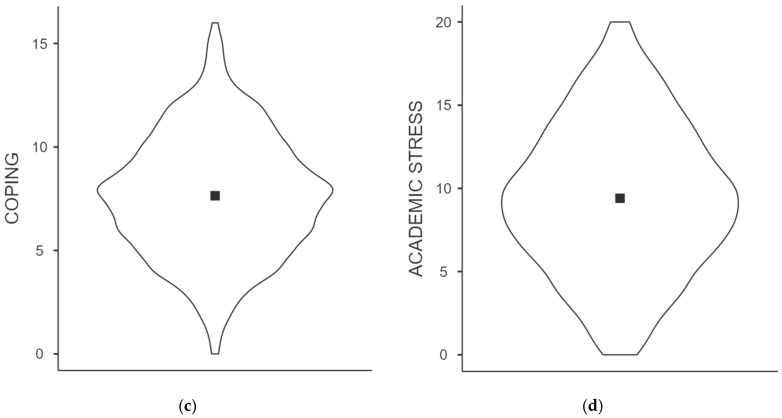
(**a**–**d**) Distribution of scores for the three dimensions of IPSS-R (Stress, Coping, and Academic Stress) and the total IPSS-R score.

Figure 1a–d present boxplots depicting the distribution of scores across the three IPSS-R dimensions—Stress, Coping, and Academic Stress—as well as the total IPSS-R score among Italian university students. Each plot displays the range, median, and variability of scores, offering a visual summary of central tendency and dispersion within the sample. The Stress dimension (Figure 1a) shows a moderate skew toward higher scores, indicating that many students experience elevated emotional strain. In contrast, the Coping dimension (Figure 1b) shows a more symmetrical distribution, suggesting diverse perceptions of stress management capabilities. Scores for Academic Stress (Figure 1c) are predominantly concentrated in the upper range, underscoring the academic environment as a major contributor to psychological pressure. The total IPSS-R scores (Figure 1d) reflect a cumulative pattern, with a substantial proportion of students scoring above the midpoint. The “midpoint” refers to the theoretical midpoint of the IPSS-R scale, corresponding to the average score that would be obtained if each item were answered with a neutral or moderate response. These trends highlight the pervasive nature of psychological distress in this population, particularly in relation to academic demands. The variability observed in Coping scores suggests that individual differences in stress regulation may play a critical role in moderating the impact of perceived stress on overall well-being.

### 3.3. Principal Component Analysis

To explore the underlying structure of stress-related experiences among university students and reduce data dimensionality, principal component analysis (PCA) was employed. The selection of components was guided by the proportion of variance explained by each component (see Table 3), while the contribution of individual items to each component is detailed in the factor loadings presented in Table 4. Based on the elbow method [29], two principal components were retained, collectively accounting for 54.6% of the total variance (Dim1: 40.9%, Dim2: 13.7%). While this level may appear modest, it is consistent with values commonly reported in psychological and social sciences, where constructs are multidimensional and influenced by multiple latent factors; in similar validation studies of stress-related instruments, explained variances between 50% and 60% are generally considered acceptable [30,31]. The difference between Component 1 and Component 2 likely reflects the stronger contribution of the primary latent dimension (overall perceived stress) relative to the secondary one (coping-related responses). This reduction enhances interpretability and provides a clearer framework for analyzing the relationship between IPSS-R scores and sociodemographic factors. The retained variables represent those contributing most significantly to the overall variance, thereby highlighting the primary dimensions of stress-related experiences. This approach strengthens both clarity and analytical rigor of subsequent examinations involving IPSS-R scores and sociodemographic characteristics.

**Figure 2:**
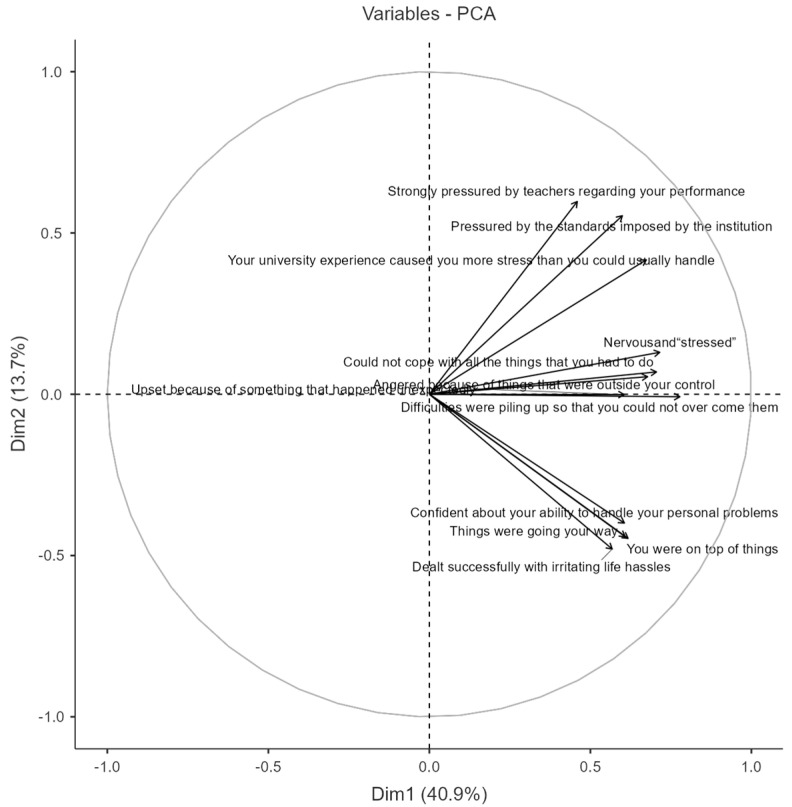
PCA plot of the IPSS-R scale variables.

The biplot in Figure 2 visualizes the projection of variables onto the first two principal components derived from the PCA. These components reveal distinct patterns in how university students experience and respond to stress. The first component (Dim1) reflects a clear stress–coping continuum. On the positive side of this axis, items such as “nervous and stressed,” “difficulties piling up so that you could not overcome them,” “pressured by the standards imposed by the institution,” and “strongly pressured by teachers regarding your performance” exhibited high loadings, indicating a cluster of experiences characterised by sustained emotional strain, high workload, and perceived external demands. Conversely, the negative side of Dim1 grouped items related to personal efficacy and control, including “confident about your ability to handle personal problems,” “you were on top of things,” and “dealt successfully with irritating life hassles,” suggesting effective coping strategies and resilience in the face of stressors.

The second component (Dim2), which explains a smaller portion of the variance, differentiates between more acute, event-specific emotional reactions and ongoing stress experiences. Items such as “upset because of something that happened unexpectedly” loaded more prominently on this axis, separating situational affective responses from the more chronic or structural sources of stress captured by Dim1.

Taken together, the PCA suggests that students’ stress experiences can be conceptualised along two main dimensions: a primary axis representing the balance between perceived stress and coping capacity, and a secondary axis distinguishing between enduring pressures (e.g., institutional or academic demands) and transient, situational emotional events. This structure provides a meaningful framework for subsequent analyses linking stress patterns to sociodemographic factors, coping resources, and academic outcomes.

### 3.4. Group Comparisons

**Figure 3:**
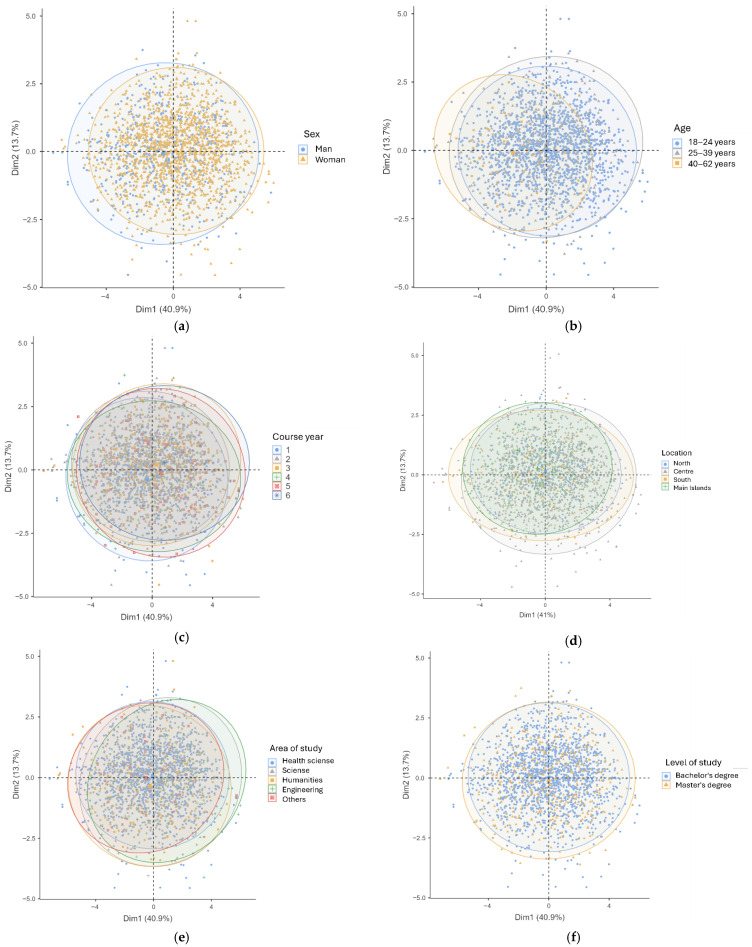

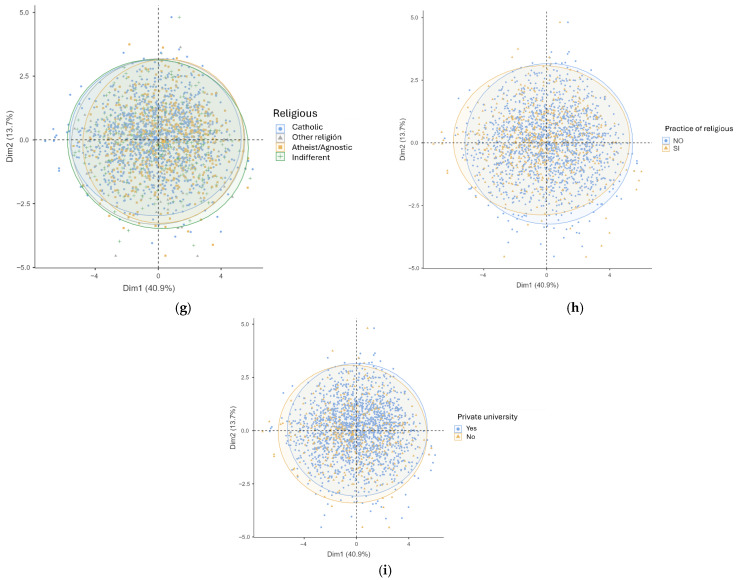
PCA plots showing participants clustering by sociodemographic variables: (**a**) sex, (**b**) age, (**c**) course year, (**d**) location, (**e**) area of study, (**f**) level of study, (**g**) religion, (**h**) religious practice, and (**i**) university type.

PCA-based group plots (Figure 3a–i) were used to explore variations in stress-related experiences across sociodemographic characteristics, with participants color-coded by category. Overall, PCA showed substantial overlap for most groups, indicating weak multivariate clustering. Mean comparisons (Supplementary Table S2a–d) revealed statistically significant differences; however, the effect sizes were generally very small, suggesting that these differences are marginal and may be influenced by a broad set of interrelated factors. Women reported higher general and academic stress and greater difficulty coping than men. Older students (40–62 years) showed lower stress, less nervousness, and better coping compared to younger groups. First-year students had higher total and academic stress scores. Geographic location had minimal impact, although students from Southern Italy and the islands exhibited slightly lower stress and better coping. Field of study influenced stress patterns, with engineering and science students reporting higher nervousness, academic stress, and coping difficulties than health sciences and humanities students. Differences by level of study and religious affiliation were small, with minor variations in specific items. Overall, sex, age, and field of study emerged as the main factors associated with variations in perceived stress and coping, but these effects appear marginal when considering their small magnitude.

## 4. Discussion

The research objectives were to investigate perceived stress profiles among Italian university students and to examine associations between sociodemographic factors, academic stress, and coping capacity. A high prevalence of perceived stress was found among Italian university students, underscoring their susceptibility to psychological distress in academic settings. Academic demands—such as workload, deadlines, and performance expectations—emerged as the primary stressors, significantly contributing to emotional strain. These findings align with international research: Olson et al. [30] identified academic workload as the leading stressor among German students, closely linked to burnout and reduced academic engagement. Similar patterns have been observed in Spain [31], the United States [11], China [32], and the Netherlands [33], where academic pressure is consistently a predictor of anxiety, depression, and poor well-being. These results support Lazarus and Folkman’s (1984) [1] Transactional Stress Model, which posits that stress arises when perceived demands exceed coping resources. Within this framework, students frequently perceive academic expectations as overwhelming, leading to heightened stress responses [1].

With respect to coping outcomes, the findings indicate that participants’ coping abilities were, on average, only moderately effective. Many students reported limited confidence in managing stress, echoing findings by Puigbó et al. (2019) and Nespereira-Campuzano & Vázquez-Campo (2017), who noted that planning strategies alone offer limited protection in the absence of emotional regulation [34,35]. Slimmen et al. (2022) similarly observed that while coping strategies can be beneficial, their effectiveness diminishes under intense academic pressure [33].

Interpersonal stressors—such as family or peer-related pressure—were comparatively less influential. This aligns with Pointon-Haas et al. (2023), who found academic stress to be more dominant, especially among students with greater autonomy [36]. However, Mize (2023) emphasized that the integration of personal coping mechanisms with institutional support enhances emotional regulation, suggesting that individual strategies alone may be insufficient [37].

Sociodemographic factors revealed notable differences. Female students reported significantly higher stress levels, particularly in relation to nervousness and academic strain, consistent with Gulick et al. [38]. In contrast, older students and those enrolled in master’s programs showed more effective coping and slightly lower stress levels, aligning with Monteiro & Balogun (2014) and Cabras et al. (2024), who linked age and experience to improved emotional regulation and resilience [39,40]. First-year students appeared especially vulnerable, likely due to the challenges associated with transitioning into university life—a trend further supported by PCA results.

Cultural and contextual factors may explain regional differences in stress and coping. Students from Southern Italy and the islands reported lower stress and more effective coping, potentially due to family cohesion and better social support networks, whereas Northern Italy is characterized by strong university competitiveness, greater performance and time pressure, a higher cost of living, and stronger individualism. This contrasts with Piumatti et al. (2016), who found higher life satisfaction in Northern Italy but greater family interdependence in the South, which may serve as a buffer against academic stress [41].

The field of study significantly influenced stress levels. Students in health sciences, science, and engineering reported higher IPSS-R scores, reflecting the impact of demanding curricula. These findings align with Pérez-Jorge et al. (2025), Olson et al. (2025), and Jensen et al. (2023), who identified STEM disciplines as particularly stress-inducing due to workload intensity and competitive environments [30,31,42]. This underscores the need for discipline-specific support strategies to mitigate stress and promote students’ well-being.

Students attending private universities reported elevated academic pressure, particularly regarding items such as “*Too much stress during university studies*” and “*Pressure for standards at school*/*university*”. This may be attributed to greater financial investment and elevated family expectations. These results are consistent with Calizaya-López et al. (2022), who found higher stress levels among Peruvian students in private institutions, and Al-Khlaiwi et al. (2024), who reported increased depression and anxiety among Saudi medical students in similar contexts [43,44]. Collectively, these findings suggest that economic and socio-familial pressures associated with private education contribute to academic stress and should be considered in institutional support planning.

Overall, the study confirms that academic pressure is the principal source of distress among university students, with significant implications for mental health and academic performance. PCA results revealed a continuum opposing control and coping abilities to stress vulnerability. Students exhibiting higher self-confidence and emotional regulation clustered in the quadrant associated with lower stress, while those experiencing academic overload and emotional reactivity clustered in the vulnerability quadrant [45,46,47].

This structure reinforces the importance of both individual and institutional interventions. At the individual level, programs aimed at enhancing self-efficacy, emotional regulation, and resilience are essential. Training in time management, mindfulness, and stress-coping strategies may reduce psychological burden [48,49,50]. Lifestyle interventions, such as regular moderate exercise, have also been shown to improve autonomic balance and cardiovascular resilience [51,52].

At the institutional level, universities should reevaluate workload distribution and assessment practices to reduce unnecessary pressure [53,54]. Structured counselling services and peer-based interventions are critical for promoting mental well-being [37]. The systematic use of validated tools like the IPSS-R can facilitate early identification of at-risk students, enabling timely and tailored support strategies [23].

These findings align with Anjala’s (2024) review, which emphasizes the multifactorial nature of academic stress, shaped by personal attributes—such as self-efficacy, resilience, problem-solving skills, and self-esteem—and contextual pressures, including family expectations, financial constraints, interpersonal relationships, and academic environments [55,56,57,58]. Integrated strategies that simultaneously strengthen individual resources and address structural determinants within higher education institutions appear particularly effective. Excessive academic pressure is associated with diminished performance, reduced satisfaction, and poorer psychological and physical health, particularly in relation to sleep quality and energy levels [59]. Promoting transversal skills, reinforcing social support systems, and fostering culturally sensitive, flexible coping approaches—ranging from recreational activities to cognitive restructuring—may therefore represent key levers to safeguard both academic success and overall student well-being [60,61]. Our findings may encourage universities to improve curriculum design; implement mental health services, support groups, and tutors trained in stress management; offer seminars on psychological health promotion and psychoeducation; and create relaxation areas.

### Strengths and Limitations

This study offers several strengths. The large, geographically diverse sample of 2103 Italian university students enhances the representativeness of the findings. The use of the IPSS-R, a validated instrument tailored to the Italian academic context, ensures accurate assessment of general stress, academic stress, and coping. The application of multivariate analyses, particularly PCA, provides a nuanced understanding of the relationships between stress dimensions and sociodemographic variables, supporting the development of targeted interventions. However, limitations must be acknowledged. The cross-sectional design precludes causal inference, and the use of convenience sampling may introduce selection bias, limiting generalizability. Reliance on self-reported data may be affected by social desirability and recall bias. Additionally, the absence of longitudinal data and objective physiological measures restricts the ability to explore stress trajectories and long-term health outcomes. Finally, self-selection bias may have led to greater participation among students interested in mental health. Moreover, online data collection may have excluded less digitally connected participants, potentially influencing regional representation. Future research should adopt longitudinal and mixed-method designs, incorporating psychophysiological assessments to validate and expand upon these findings.

## 5. Conclusions

This study highlights substantial levels of perceived stress among Italian university students, alongside notable variability across sociodemographic groups. Key correlates of stress included academic workload, coping capacity, and stress-related experiences within the university environment. These findings indicate the importance of monitoring stress in student populations and support the value of using validated instruments such as the IPSS-R to identify groups at heightened risk. Universities may wish to consider strategies to promote students’ well-being based on these results, including reviewing academic demands in more intensive programs and strengthening support systems for students with lower coping resources. Continued research, particularly longitudinal and intervention studies using the IPSS-R, would be useful to deepen understanding of stress dynamics and to evaluate initiatives aimed at fostering healthier learning conditions and safeguarding academic performance.

## Figures and Tables

**Table 1:** Sample sociodemographic characteristics (N = 2103).

**Sex**	**N**	**%**
Male	500	23.80%
Female	1603	76.20%
**Age**	**Mean**	**SD**
	23.4	5.68
	**N**	**%**
Young (18–24 years)	1608	76.46%
Young adults (25–39 years)	431	20.45%
Middle age (40–62 years)	65	3.09%
**Religious Faith**	**N**	**%**
Roman catholic	1103	52.40%
Other religion	137	6.50%
Atheist/Agnostic	571	27.20%
Indifferent	292	13.90%
**Religious Practice**	**N**	**%**
No	1496	71.10%
Yes	607	28.90%
**Study Level**	**N**	**%**
Bachelor’s degree	1653	78.60%
Master’s degree	450	21.40%
**Course of Studies**	**N**	**%**
Health science (Nursing, Medicine, Dentistry, Nutrition, Psychology)	1477	70.20%
Science (Mathematics, Physics, Chemistry, Biology, Biotechnology, etc.)	122	5.80%
Humanities (Philosophy, Literature, Communication, Law, Educational sciences, Economics, etc.)	275	13.10%
Engineering	175	8.30%
Other	54	2.60%
**Course Year**	**N**	**%**
1	488	23.20%
2	881	41.90%
3	396	18.80%
4	120	5.70%
5	147	7.00%
6	71	3.40%
**Geographical Area**	**N**	**%**
North	330	15.70%
Centre	1157	55.00%
South	156	7.40%
Main islands	460	21.90%
**Private University**	**N**	**%**
No	1717	81.60%
Yes	386	18.40%
**Offsite**	**N**	**%**
No	1145	64.18%
Yes	638	35.76%
No answer	320	17.94%
**Scholarship/Place in the College of Merit**	**N**	**%**
No	1431	80.62%
Yes	344	19.38%
No answer	328	18.48%

Legend: SD = standard deviation.

**Table 2:** Scores of IPSS-R questionnaire items and factors (N = 2103).

	Mean	SD		
Upset because of something that happened unexpectedly	1.61	1.09		
Unable to control the important things in your life	1.68	1.10		
Nervous and “stressed”	3.08	0.91		
Could not cope with all the things that you had to do	2.47	1.05		
Angered because of things that were outside your control	2.32	1.13		
Difficulties were piling up so that you could not overcome them	1.73	1.20		
Confident about your ability to handle your personal problems	1.59	0.96		
Things were going your way	2.05	0.90		
Dealt successfully with irritating life hassles	1.90	0.89		
You were on top of things	2.11	0.97		
Pressured by the standards imposed by the institution	2.84	1.11		
Strongly pressured by teachers regarding your performance	1.69	1.29		
Competition with classmates about grades was very intense	1.30	1.32		
Strongly pressured by your family about your grades	1.18	1.31		
Your university experience caused you more stress than you could usually handle	2.38	1.22		
STRESS	12.90	4.86	2.15 ^	0.81 ^
COPING	7.64	2.90	1.91 ^	0.73 ^
ACADEMIC STRESS	9.41	4.46	1.88 ^	0.89 ^
IPSS-R	29.90	9.94	1.99 ^	0.66 ^

Legend: SD = standard deviation; ^ = weighted value for the number of items.

**Table 3:** PCA’s percentage of explained variance.

Component		Eigenvalue	Percentage of Variance
1	Things were going your way	4.905	40.87
2	Your university experience caused you more stress than you could usually handle	1.647	13.72
3	Dealt successfully with irritating life hassles	0.999	8.32
4	You were on top of things	0.678	5.65
5	Pressured by the standards imposed by the institution	0.612	5.10
6	Confident about your ability to handle your personal problems	0.581	4.84
7	Nervous and “stressed”	0.525	4.37
8	Difficulties were piling up so that you could not overcome them	0.489	4.08
9	Could not cope with all the things that you had to do	0.434	3.62
10	Angered because of things that were outside your control	0.415	3.46
11	Strongly pressured by teachers regarding your performance	0.367	3.06
12	Upset because of something that happened unexpectedly	0.348	2.90

**Table 4:** Contribution of individual items to each component.

	Component		
	1	2	Uniqueness
You were on top of things	0.754		0.419
Things were going your way	0.744		0.435
Dealt successfully with irritating life hassles	0.742		0.447
Confident about your ability to handle your personal problems	0.712		0.474
Difficulties were piling up so that you could not overcome them	0.561	0.539	0.395
Upset because of something that happened unexpectedly	0.433	0.422	0.634
Pressured by the standards imposed by the institution		0.815	0.334
Your university experience caused you more stress than you could usually handle		0.769	0.372
Strongly pressured by teachers regarding your performance		0.748	0.432
Nervous and “stressed”	0.420	0.593	0.471
Could not cope with all the things that you had to do	0.456	0.543	0.498
Angered because of things that were outside your control	0.446	0.513	0.538

## Data Availability

The data presented in this study are available from the corresponding author upon reasonable request. The data are not publicly available due to privacy and ethical restrictions.

## Supplementary Materials

The following supporting information can be downloaded at: https://www.mdpi.com/article/10.3390/healthcare13222830/s1, Table S1: Items of the Italian Perceived Stress Scale—Revised (IPSS-R); Table S2a: Comparison between IPSS-R scores, sample’s age and study site; Table S2b: Comparison between IPSS-R scores and sample’s variables sex, religious affiliation and practice; Table S2c: Comparison between IPSS-R scores and sample’s variables area and level of study; Table S2d: Comparison between IPSS-R scores and sample’s contextual academic variables.

## Abbreviations

The following abbreviations are used in this manuscript:

IPSS-R	Italian Perceived Stress Scale—Revised
PSS	Perceived Stress Scale
PCA	Principal Component Analysis
STROBE	Strengthening the Reporting of Observational Studies in Epidemiology
HPA	Hypothalamic–Pituitary–Adrenal (axis)
SAM	Sympathetic–Adrenal–Medullary (system)
STEM	Science, Technology, Engineering, and Mathematics
SD	Standard Deviation
IRB	Institutional Review Board
CECRI	Center of Excellence for Nursing Scholarship (Rome, Italy)
