# Supplementary material for: Perceived Stress Profiles Among Italian University Students: A Multivariate Approach
Source: Healthcare (Basel). 2025 Nov 7;13(22):2830. doi: 10.3390/healthcare13222830 (PMC12652295; doi:10.3390/healthcare13222830)
Supplement: Supplementary file 1 [file healthcare-13-02830-s001.zip › healthcare-3899733-supplementary.pdf]

Table S1. Items of the Italian Perceived Stress-Revised Scale (IPSS-R)

IPSS-R1. Upset because of something that happened unexpectedly?
IPSS-R2. Unable to control the important things in your life?
IPSS-R3. Nervous and “stressed”?
IPSS-R4. Could not cope with all the things that you had to do?
IPSS-R5. Angered because of things that were outside your control?
IPSS-R6. Difficulties were piling up so that you could not overcome them?
IPSS-R7. Confident about your ability to handle your personal problems?
IPSS-R8. Things were going your way?
IPSS-R9. Dealt successfully with irritating life hassles?
IPSS-R10. You were on top of things?
IPSS-R11. Pressured by the standards imposed by the institution (school/university)?
IPSS-R12. Strongly pressured by teachers regarding your performance?
IPSS-R13. Competition with classmates about grades was very intense?
IPSS-R14. Strongly pressured by your family about your grades?
IPSS-R15. Your university experience caused you more stress than you could usually handle?

Table S2.a. Comparison between IPSS-R scores, sample's age and study site.

	Whole Sample		Age								Location									
			Young		Young adults		Middle age				North		Centre		South		Islands			
	**Mean**	**SD**	**Mean**	**SD**	**Mean**	**SD**	**Mean**	**SD**	**p-value&**	**ε²**	**Mean**	**SD**	**Mean**	**SD**	**Mean**	**SD**	**Mean**	**SD**	**p-value&**	**ε²**
Upset because of something that happened unexpectedly	1.61	1.09	1.63	1.07	1.62	1.11	1.00	0.99	<0.001*	0.009	1.60	1.00	1.66	1.10	1.44	1.17	1.54	1.07	0.053	0.004
Unable to control the important things in your life	1.68	1.10	1.69	1.09	1.73	1.12	1.23	1.05	0.002*	0.006	1.63	1.04	1.79	1.13	1.58	1.11	1.47	1.03	<0.001*	0.014
Nervousand"stressed"	3.08	0.91	3.12	0.88	3.04	0.95	2.25	0.94	<0.001*	0.024	2.99	0.89	3.16	0.89	3.01	0.98	2.97	0.93	<0.001*	0.011
Could not cope with all the things that you had to do	2.47	1.05	2.47	1.03	2.53	1.09	2.06	0.96	0.002*	0.006	2.53	0.97	2.55	1.07	2.38	1.07	2.23	0.99	<0.001*	0.017
Angered because of things that were outside your control	2.32	1.13	2.36	1.11	2.30	1.17	1.61	1.00	<0.001*	0.013	2.36	1.03	2.40	1.14	2.17	1.19	2.15	1.13	<0.001*	0.009
Difficulties were piling up so that you could not over come them	1.73	1.20	1.73	1.19	1.79	1.22	1.11	0.95	<0.001*	0.009	1.78	1.07	1.82	1.26	1.69	1.18	1.47	1.08	<0.001*	0.013
Confident about your ability to handle your personal problems	1.59	0.96	1.62	0.95	1.53	0.99	1.05	0.84	<0.001*	0.012	1.65	0.91	1.65	0.98	1.56	0.97	1.39	0.91	<0.001*	0.011
Things were going your way	2.05	0.90	2.07	0.90	2.02	0.92	1.64	0.70	<0.001*	0.007	2.03	0.80	2.13	0.93	2.01	0.94	1.86	0.86	<0.001*	0.014
Dealt successfully with irritating life hassles	1.90	0.89	1.94	0.89	1.80	0.85	1.50	0.80	<0.001*	0.012	1.82	0.83	2.01	0.92	1.79	0.91	1.73	0.78	<0.001*	0.018
You were on top of things	2.11	0.97	2.14	0.96	2.09	1.00	1.59	0.92	<0.001*	0.009	2.04	0.90	2.24	1.01	2.03	0.96	1.87	0.87	<0.001*	0.025
Pressured by the standards imposed by the institution	2.84	1.11	2.86	1.09	2.86	1.13	2.11	0.98	<0.001*	0.016	2.99	0.94	2.77	1.18	2.69	1.13	2.96	0.99	0.007*	0.006
Strongly pressured by teachers regarding your performance	1.69	1.29	1.69	1.27	1.75	1.37	1.41	1.18	0.165	0.002	1.82	1.22	1.64	1.35	1.69	1.25	1.72	1.18	0.071	0.003
Competition with classmates about grades was very intense	1.30	1.32	1.32	1.31	1.33	1.36	0.63	0.93	<0.001*	0.009	1.44	1.29	1.40	1.38	1.04	1.21	1.05	1.16	<0.001*	0.014
Strongly pressured by your family about your grades	1.18	1.31	1.25	1.33	1.06	1.25	0.45	0.96	<0.001*	0.015	1.35	1.35	1.26	1.34	1.02	1.22	0.92	1.19	<0.001*	0.014
Your university experience caused you more stress than you could usually handle	2.38	1.22	2.41	1.18	2.45	1.30	1.44	1.13	<0.001*	0.019	2.55	1.11	2.37	1.27	2.14	1.23	2.38	1.12	0.009*	0.006
STRESS	12.90	4.86	13.00	4.74	13.00	5.16	9.27	4.16	<0.001*	0.018	12.90	4.39	13.40	5.02	12.30	5.08	11.80	4.47	<0.001*	0.017
COPING	7.64	2.90	7.77	2.86	7.45	2.96	5.78	2.54	<0.001*	0.015	7.54	2.56	8.03	3.02	7.39	3.09	6.85	2.56	<0.001*	0.026
ACADEMIC STRESS	9.41	4.46	9.53	4.35	9.45	4.80	6.03	3.52	<0.001*	0.019	10.20	3.94	9.45	4.83	8.58	4.07	9.04	3.85	<0.001*	0.008
**IPSS-R**	**30.60**	**7.08**	**30.30**	**9.67**	**29.90**	**10.50**	**21.10**	**8.15**	**<0.001***	**0.025**	**30.60**	**8.86**	**30.90**	**10.50**	**28.20**	**10.20**	**27.70**	**8.76**	**<0.001***	**0.018**

*Legend: SD= Standard Deviation; &= Kruskal Wallis test → ε² (epsilon squared); *= Statistically significant.*

Table S2.b. Comparison between IPSS-R scores and sample's variables sex, religious affiliation and practice.

	Religion										Practice of their religion						Sex					
	Catholic		Other religion		Atheist/Agnostic		Indifferent				No		Yes				Man		Woman			
	Mean	SD	Mean	SD	Mean	SD	Mean	SD	p-value&	ε²	Mean	SD	Mean	SD	p-value$	*effect size r*	Mean	SD	Mean	SD	p-value$	*effect size r*
Upset because of something that happened unexpectedly	1.56	1.09	1.69	1.10	1.69	1.08	1.57	1.09	0.111	0.003	1.63	1.08	1.56	1.09	0.251	0.001	1.49	1.08	1.64	1.08	0.009*	0.003
Unable to control the important things in your life	1.58	1.09	1.79	1.12	1.84	1.09	1.72	1.12	<0.001*	0.010	1.72	1.10	1.58	1.09	0.003*	0.004	1.64	1.13	1.69	1.09	0.405	0.000
Nervousand“stressed”	3.04	0.93	3.00	1.05	3.16	0.83	3.12	0.91	0.121	0.003	3.13	0.87	2.96	0.98	<0.001*	0.005	2.76	1.04	3.18	0.84	<0.001*	0.030
Could not cope with all the things that you had to do	2.39	1.04	2.55	1.06	2.59	1.04	2.48	1.05	0.003*	0.007	2.52	1.04	2.34	1.06	<0.001*	0.006	2.28	1.10	2.53	1.02	<0.001*	0.009
Angered because of things that were outside your control	2.23	1.15	2.27	1.11	2.51	1.04	2.31	1.18	<0.001*	0.011	2.38	1.12	2.18	1.13	<0.001*	0.006	2.02	1.18	2.42	1.10	<0.001*	0.020
Difficulties were piling up so that you could not over come them	1.65	1.18	1.74	1.17	1.86	1.19	1.75	1.25	0.007*	0.006	1.78	1.20	1.60	1.19	0.004*	0.004	1.55	1.22	1.78	1.18	<0.001*	0.007
Confident about your ability to handle your personal problems	1.53	0.95	1.55	1.04	1.66	0.93	1.66	1.00	0.030*	0.004	1.63	0.96	1.47	0.95	<0.001*	0.006	1.47	1.01	1.62	0.94	0.001*	0.005
Things were going your way	2.00	0.91	2.05	0.88	2.14	0.87	2.02	0.94	0.013*	0.005	2.08	0.90	1.96	0.90	0.006*	0.004	1.91	0.93	2.09	0.89	<0.001*	0.009
Dealt successfully with irritating life hassles	1.86	0.88	1.93	0.95	1.97	0.85	1.91	0.96	0.105	0.003	1.95	0.88	1.79	0.89	<0.001*	0.006	1.82	0.93	1.93	0.87	0.009*	0.003
You were on top of things	2.02	0.95	2.16	1.03	2.24	0.94	2.17	1.04	<0.001*	0.011	2.16	0.97	1.99	0.97	<0.001*	0.007	1.98	1.00	2.15	0.96	<0.001*	0.006
Pressured by the standards imposed by the institution	2.84	1.06	2.81	1.06	2.88	1.17	2.78	1.15	0.287	0.002	2.87	1.12	2.77	1.06	0.017*	0.003	2.57	1.20	2.92	1.06	<0.001*	0.016
Strongly pressured by teachers regarding your performance	1.73	1.28	1.69	1.33	1.75	1.32	1.42	1.23	0.002*	0.007	1.67	1.30	1.74	1.26	0.192	0.001	1.64	1.34	1.71	1.27	0.209	0.001
Competition with classmates about grades was very intense	1.33	1.32	1.42	1.34	1.30	1.33	1.14	1.26	0.101	0.003	1.29	1.32	1.33	1.31	0.517	0.000	1.07	1.27	1.38	1.32	<0.001*	0.011
Strongly pressured by your family about your grades	1.12	1.29	1.26	1.32	1.33	1.34	1.12	1.33	0.011*	0.005	1.22	1.33	1.09	1.28	0.044	0.002	1.21	1.30	1.18	1.32	0.514	0.000
Your university experience caused you more stress than you could usually handle	2.35	1.19	2.31	1.29	2.49	1.22	2.36	1.26	0.101	0.003	2.43	1.21	2.28	1.22	0.010*	0.003	2.15	1.26	2.46	1.19	<0.001*	0.011
STRESS	12.40	4.82	13.10	5.17	13.70	4.63	13.00	5.10	<0.001*	0.010	13.20	4.78	12.20	4.97	<0.001*	0.007	11.70	5.02	13.20	4.75	<0.001*	0.016
COPING	7.41	2.89	7.69	2.89	8.02	2.74	7.76	3.13	<0.001*	0.008	7.82	2.89	7.21	2.88	<0.001*	0.010	7.18	3.03	7.79	2.84	<0.001*	0.008
ACADEMIC STRESS	9.37	4.33	9.47	4.42	9.75	4.68	8.82	4.49	0.023*	0.005	9.48	4.51	9.21	4.32	0.171	0.001	8.63	4.54	9.65	4.41	<0.001*	0.009
**IPSS-R**	**29.20**	**9.99**	**30.20**	**9.73**	**31.40**	**9.47**	**29.50**	**10.40**	**<0.001***	**0.009**	**30.50**	**9.80**	**28.60**	**10.20**	**<0.001***	**0.006**	**27.60**	**10.20**	**30.70**	**9.74**	**<0.001***	**0.016**

*Legend: SD= Standard Deviation; $= Mann–Whitney-U test → effect size r; &= Kruskal Wallis test → ε² (epsilon squared); *= Statistically significant.*

Table S2.c. Comparison between IPSS-R scores and sample's variables area and level of study.

	Area of study												Level of study					
	Health science		Science		Humanities		Engineering		Others				Bachelor's degree		Master's degree			*effect size r*
	Mean	SD	Mean	SD	Mean	SD	Mean	SD	Mean	SD	p-value&	ε^2	Mean	SD	Mean	SD	p-value$	
Upset because of something that happened unexpectedly	1.57	1.09	1.75	1.10	1.67	1.04	1.71	1.15	1.59	1.06	0.198	0.003	1.62	1.08	1.57	1.12	0.201	0.001
Unable to control the important things in your life	1.61	1.09	1.94	1.09	1.74	1.12	2.00	1.14	1.59	1.02	<0.001*	0.014	1.68	1.10	1.70	1.11	0.716	0.000
Nervousand"stressed"	3.03	0.92	3.31	0.89	3.09	0.88	3.29	0.80	3.06	0.92	<0.001*	0.011	3.08	0.91	3.10	0.92	0.587	0.000
Could not cope with all the things that you had to do	2.44	1.03	2.64	1.03	2.39	1.08	2.81	1.01	2.07	1.11	<0.001*	0.015	2.46	1.03	2.49	1.10	0.551	0.000
Angered because of things that were outside your control	2.29	1.13	2.39	1.12	2.36	1.17	2.49	1.03	2.33	1.13	0.255	0.003	2.33	1.13	2.30	1.12	0.440	0.000
Difficulties were piling up so that you could not over come them	1.69	1.18	1.93	1.22	1.62	1.20	2.09	1.22	1.52	1.22	<0.001*	0.011	1.73	1.17	1.72	1.28	0.758	0.000
Confident about your ability to handle your personal problems	1.55	0.95	1.73	0.98	1.56	0.96	1.84	0.99	1.41	0.88	0.002*	0.008	1.59	0.96	1.56	0.97	0.604	0.000
Things were going your way	2.00	0.88	2.26	0.97	2.02	0.95	2.35	0.88	1.89	0.90	<0.001*	0.015	2.04	0.89	2.07	0.93	0.596	0.000
Dealt successfully with irritating life hassles	1.85	0.86	1.98	0.95	1.95	0.91	2.21	0.93	1.80	0.90	<0.001*	0.012	1.89	0.88	1.94	0.92	0.475	0.000
You were on top of things	2.06	0.95	2.33	1.07	2.08	0.96	2.46	0.99	1.91	0.92	<0.001*	0.016	2.08	0.96	2.22	1.02	0.007*	0.004
Pressured by the standards imposed by the institution	2.89	1.06	2.92	1.14	2.52	1.21	2.98	1.17	2.44	1.19	<0.001*	0.016	2.87	1.09	2.74	1.16	0.040	0.002
Strongly pressured by teachers regarding your performance	1.74	1.24	1.58	1.48	1.33	1.27	1.96	1.44	1.72	1.32	<0.001*	0.016	1.70	1.27	1.68	1.36	0.633	0.000
Competition with classmates about grades was very intense	1.31	1.31	1.20	1.28	1.17	1.32	1.50	1.38	1.37	1.34	0.089	0.004	1.23	1.27	1.58	1.43	<0.001*	0.009
Strongly pressured by your family about your grades	1.15	1.29	1.17	1.29	1.26	1.40	1.44	1.42	0.85	1.12	0.041	0.005	1.19	1.33	1.16	1.26	0.872	0.000
Your university experience caused you more stress than you could usually handle	2.40	1.18	2.52	1.32	2.00	1.26	2.82	1.20	2.07	1.15	<0.001*	0.026	2.39	1.20	2.36	1.26	0.583	0.000
STRESS	12.60	4.81	14.00	4.88	12.90	4.97	14.40	4.79	12.20	4.45	<0.001*	0.013	12.90	4.80	12.90	5.05	0.836	0.000
COPING	7.48	2.82	8.30	3.01	7.61	3.06	8.86	2.90	7.00	2.73	<0.001*	0.022	7.60	2.87	7.79	2.99	0.203	0.001
ACADEMIC STRESS	9.50	4.26	9.39	4.80	8.29	4.81	10.70	4.88	8.46	4.50	<0.001*	0.016	9.38	4.35	9.51	4.83	0.736	0.000
**IPSS-R**	**29.60**	**9.67**	**31.60**	**10.20**	**28.80**	**10.30**	**34.00**	**10.40**	**27.60**	**9.53**	**<0.001***	**0.019**	**29.90**	**9.79**	**30.20**	**10.50**	**0.639**	**0.000**

*Legend: SD= Standard Deviation; $= Mann–Whitney-U test → effect size r; &= Kruskal Wallis test → ε² (epsilon squared); *= Statistically significant.*

Table S2.d. Comparison between IPSS-R scores and sample's contextual academic variables.

	Private University						Off Site						Scholarship/place in the College of Merit					
	**No**		**Yes**			*effect size r*	**No**		**Yes**			*effect size r*	**No**		**Yes**			*effect size r*
	**Mean**	**SD**	**Mean**	**SD**	**p-value$**		**Mean**	**SD**	**Mean**	**SD**	**p-value$**		**Mean**	**SD**	**Mean**	**SD**	**p-value$**	
Upset because of something that happened unexpectedly	1.66	1.08	1.36	1.08	<0.001*	0.013	1.65	1.07	1.63	1.11	0.802	0.006	1.65	1.09	1.61	1.05	0.727	0.006
Unable to control the important things in your life	1.71	1.10	1.57	1.10	0.027	0.002	1.69	1.07	1.70	1.16	0.890	0.001	1.73	1.11	1.58	1.06	0.031	0.004
Nervousand"stressed"	3.11	0.90	2.96	0.95	0.007*	0.003	3.12	0.87	3.07	0.95	0.620	0.002	3.10	0.90	3.09	0.90	0.799	0.001
Could not cope with all the things that you had to do	2.46	1.05	2.48	1.05	0.840	0.000	2.46	1.03	2.45	1.07	0.705	0.001	2.50	1.04	2.27	1.04	<0.001*	0.007
Angered because of things that were outside your control	2.35	1.12	2.21	1.18	0.070	0.002	2.35	1.11	2.30	1.16	0.396	0.000	2.34	1.14	2.30	1.08	0.443	0.000
Difficulties were piling up so that you could not over come them	1.75	1.18	1.63	1.25	0.074	0.002	1.74	1.17	1.74	1.20	0.851	0.001	1.78	1.19	1.53	1.15	<0.001*	0.007
Confident about your ability to handle your personal problems	1.59	0.95	1.55	1.01	0.538	0.000	1.59	0.94	1.58	0.96	0.930	0.000	1.62	0.95	1.43	0.93	0.002*	0.006
Things were going your way	2.06	0.90	1.98	0.90	0.134	0.001	2.06	0.89	2.04	0.93	0.592	0.000	2.07	0.91	1.98	0.88	0.168	0.002
Dealt successfully with irritating life hassles	1.90	0.87	1.93	0.94	0.362	0.000	1.90	0.87	1.87	0.89	0.128	0.002	1.91	0.88	1.81	0.83	0.068	0.003
You were on top of things	2.11	0.96	2.11	1.03	0.901	0.000	2.12	0.95	2.06	0.97	0.202	0.002	2.12	0.96	1.99	0.92	0.022*	0.004
Pressured by the standards imposed by the institution	2.89	1.08	2.62	1.17	<0.001*	0.008	2.83	1.10	2.97	1.05	0.008*	0.009	2.88	1.08	2.86	1.09	0.764	0.005
Strongly pressured by teachers regarding your performance	1.71	1.29	1.61	1.26	0.182	0.001	1.66	1.29	1.79	1.28	0.029	0.003	1.71	1.31	1.66	1.23	0.584	0.000
Competition with classmates about grades was very intense	1.25	1.29	1.52	1.41	<0.001*	0.005	1.26	1.29	1.22	1.29	0.467	0.008	1.29	1.29	1.08	1.25	0.003*	0.011
Strongly pressured by your family about your grades	1.19	1.33	1.16	1.24	0.856	0.000	1.20	1.33	1.16	1.31	0.588	0.000	1.24	1.33	0.93	1.27	<0.001*	0.009
Your university experience caused you more stress than you could usually handle	2.43	1.21	2.17	1.23	<0.001*	0.007	2.40	1.23	2.46	1.18	0.308	0.005	2.44	1.23	2.35	1.12	0.151	0.006
STRESS	13.00	4.81	12.20	5.01	0.003*	0.004	13.00	4.69	12.90	5.04	0.645	0.001	13.10	4.86	12.40	4.63	0.014*	0.004
COPING	7.86	2.86	7.58	3.03	0.685	0.000	7.67	2.83	7.54	2.95	0.408	0.001	7.72	2.89	7.21	2.72	0.005*	0.005
ACADEMIC STRESS	9.48	4.43	9.09	4.59	0.091	0.001	9.35	4.44	9.61	4.33	0.199	0.001	9.57	4.49	8.88	4.05	0.010*	0.003
**IPSS-R**	**30.20**	**9.82**	**28.90**	**10.40**	**0.017***	**0.003**	**30.00**	**9.58**	**30.00**	**10.20**	**0.597**	**0.000**	**30.40**	**9.95**	**28.50**	**9.21**	**0.003***	**0.006**

*Legend: SD= Standard Deviation; $= Mann–Whitney-U test → effect size r; &= Kruskal Wallis test → ε² (epsilon squared); *= Statistically significant.*
